# Provision of the levonorgestrel intrauterine system in Nigeria: Provider perspectives and service delivery costs

**DOI:** 10.12688/gatesopenres.13135.1

**Published:** 2020-08-06

**Authors:** Aurélie Brunie, Kate H. Rademacher, Anthony Adindu Nwala, Kendal Danna, Mariya Saleh, Kayode Afolabi

**Affiliations:** 1 FHI 360, Washington DC, North Carolina, USA; 2 FHI 360, Durham, North Carolina, USA; 3 Society for Family Health, Abuja, Nigeria; 4 PSI, Washington DC, USA; 5 Chemonics, Abuja, Nigeria; 6 Federal Ministry of Health, Abuja, Nigeria

**Keywords:** contraception, long-acting reversible contraceptive, levonorgestrel intrauterine system

## Abstract

**Background**: Several organizations in Nigeria are leading pilot introduction programs of the levonorgestrel intrauterine system (LNG-IUS). We conducted a qualitative assessment of providers’ experiences across the five programs and an analysis of service delivery costs in one program.

**Methods**: We conducted 20 in-depth interviews (IDIs) with providers. We used project expenditure records to estimate incremental direct service delivery costs of introducing the LNG-IUS in 40 social franchise clinics supported by the Society for Family Health (SFH). We then compared the direct service delivery costs per couple years of protection (CYP) for the LNG-IUS to other family planning methods.

**Results**: Providers appreciated the therapeutic benefits of the LNG-IUS, especially reduction of heavy bleeding. They said that women generally accepted bleeding changes with counseling but noted complaints about spotting and mixed acceptability of amenorrhea. Providers indicated being comfortable with both the insertion and removal process and believed their equipment and infection prevention protocols were adequate. Lack of awareness among women, limited availability, current pricing, and resistance to uterine placement among some women were perceived as barriers. The estimated direct service delivery cost of introducing the LNG-IUS in pilot settings, inclusive of up-front provider training costs, was USD 34 per insertion. Direct service delivery costs at a ‘steady state’ (i.e., without training costs included for any method) of the LNG-IUS per CYP was similar to that of other contraceptive methods distributed in Nigeria.

**Conclusion**: Providers’ positive experiences with the LNG-IUS and direct service delivery costs per CYP that align with those for other methods suggest that the LNG-IUS could be an important addition to the method mix in Nigeria. Product introduction strategies will need to address both the supply and the demand sides, as well as consider appropriate pricing of the LNG-IUS relative to other methods and particularly the copper IUD.

## Introduction

In addition to being one of the most effective forms of reversible contraception available, the levonorgestrel intrauterine system (LNG-IUS) can reduce the duration and amount of menstrual bleeding and can result in fewer systemic side effects than other hormonal methods. The method is a proven treatment for menorrhagia, and it can be used to manage uterine fibroids and endometriosis and potentially to alleviate anemia ^1– 4^. Approved for general public use in the 1990s in Europe and in 2000 in the United States, the innovator product (Mirena ^®^) enjoys considerable success in these markets, with approximately 74 percent of all intrauterine contraceptive users in the United States using a hormonal IUS product (versus 26 percent using a copper product) ^5^. Yet 30 years after initial product introduction, the method’s availability in low- and middle-income countries (LMICs) remains very limited, in part due to high commodity costs. To date, the LNG-IUS has not been included in any tenders issued by major international donor agencies for use in the public sector in LMICs ^4^.

Since 2005, the International Contraceptive Access (ICA) Foundation has been donating limited quantities of free, unbranded LNG-IUS devices for distribution. A number of countries, including Nigeria, have used this donated product to support pilot introduction activities ^6^. More recently, new, more affordable commercial LNG-IUS products have received regulatory approval in (see Table 1), suggesting there may be opportunities in coming years to further expand access to the method within the country ^7^.

**Table 1.** Overview of levonorgestrel intrauterine system (LNG-IUS) products that have received regulatory approval in Nigeria (as of April 2020).

Supplier	Product	Registered in Nigeria	Overview of availability in Nigeria
Bayer Healthcare	Mirena® *	Yes	Mirena is provided commercially through private healthcare clinics on a limited basis.
International Contraceptive Access (ICA) Foundation	Unbranded LNG-IUS product	Yes	Through a public-private partnership between Bayer HealthCare & Population Council, a free unbranded LNG-IUS product is donated by application for small-scale, pilot activities. Programs are allowed to charge up to USD 10 to clients as a fee for insertion and removal services.
Medicines360	AVIBELA®	Yes	Sold in the U.S. under trade name LILETTA®. After review by the National Agency for Food and Drug Administration and Control (NAFDAC) notification of regulatory approval as of December 2019 under the trade name AVIBELA. The public sector price to distributors for AVIBELA will vary by volume between USD 12-16; for an order of 100,000 units, public sector transfer price will be approximately USD 15/unit ^7^.
Pregna	Eloira	Yes	Eloira is manufactured by Pregna International Ltd. based in India and distributed in Nigeria by DKT ^7^.
HLL Lifecare Ltd.	Emily	Yes	Emily is manufactured by HLL Lifecare based in India. Product consists of a small white M-shaped frame which contains levonorgestrel; this differs from the other LNG-IUS products which are T-shaped ^7^.

* Bayer Healthcare also manufactures the LNG-IUS products Skyla and Kyleena. However, these products are not yet available in Nigeria and therefore are not discussed here.

Service providers are an important gateway to the use of many contraceptives, and in particular long-acting reversible methods which require special training and skills for insertion and removal. Providers are often a trusted source of information about method selection and are well-positioned to alleviate concerns among women through counseling, including on side effects ^8– 11^. At the same time, providers can create barriers to access which can include inadequate or inaccurate counseling or poor service provision ^12, 13^. Given their role, it is important to understand providers’ perspectives and experiences with the LNG-IUS to identify potential barriers and opportunities to introducing and scaling-up the method. In addition, decision-makers such as program managers, governments and donors need information on the incremental service delivery costs (inclusive of provider training, demand creation, and supplies for routine service delivery) associated with adding the LNG-IUS to the method mix ^14^.

Although the copper intrauterine device (IUD) and LNG-IUS have distinct features, common traits between the methods that have contributed to low uptake of the copper IUD in many developing countries could have implications for providers’ attitudes and behaviors regarding LNG-IUS provision ^13^. These include required skill levels for insertion (which can furthermore be difficult to maintain in contexts with low client demand); uterine placement (with concerns among providers related to infections or risks of infertility and misunderstandings leading to biases against IUD use particularly among nulliparous women); and the fact that inserting IUDs is more time-consuming than provision of implants or short-acting methods ^15^. To date, evidence on provider perspectives specific to the LNG-IUS is encouraging but limited. Qualitative interviews with 12 public sector providers in Ghana showed that providers were generally satisfied with the product and found it easy to insert and remove, although half would have liked additional training ^16^. A survey of 27 providers from Marie Stopes Kenya ^17^ and qualitative interviews with 32 providers in the Marie Stopes International Organisation Nigeria (MSION) service network ^14^ both revealed positive attitudes towards the LNG-IUS and positive insertion experiences, though some providers were less comfortable providing the LNG-IUS compared to the subdermal implant or copper IUD or initially experienced some challenges. In addition to lacking evidence from more contexts and service delivery channels, additional insights into broader introduction strategies inclusive of provider work settings and opportunities created through demand-generation and pricing strategies are needed to more fully understand barriers and opportunities to introducing the LNG-IUS.

As of 2019, several groups in Nigeria, including DKT International, MSION, Rotary International, the Society for Family Health (SFH) and the University College Hospital Ibadan (UCHI) had introduced the LNG-IUS in their programs, either using units donated by the ICA Foundation (four programs) or Eloira, a product manufactured by Pregna in India (distributed by DKT). The range of service delivery approaches, pricing strategies and accompanying demand-generation efforts offer an opportunity to generate learnings to support decisions related to future introduction and scale-up of the LNG-IUS in Nigeria. This paper also offers an analysis of the incremental service delivery costs of introducing the LNG-IUS in the SFH program, and an analysis of the direct service delivery costs per couple years of protection (CYP) of the LNG-IUS compared to other family planning methods.

## Methods

### Setting

In Nigeria, modern contraceptive use among all women stagnated around 11% between 2008 and 2013 ^18, 19^. Data from the 2018 Performance Monitoring & Accountability (PMA) 2020 survey found a modern contraceptive prevalence of 18%, suggesting some gains may have been made more recently, although the survey only spanned seven of Nigeria’s 36 states ^20^. Available estimates also highlight regional disparities, with more limited use of modern contraception in the North of the country ^19, 20^.

In recent years, several implementing partners have successfully increased provision of implants and copper IUDs in Nigeria as part of a broader effort by the government and non-profit partners to increase access to long-acting reversible contraceptives (LARCs) and overall contraceptive prevalence ^21^. In the PMA2020 survey, implants and IUDs accounted for 24% and 6% of the method mix among married women, respectively, but less than 3% of unmarried sexually active contraceptive users used each of these methods ^20^. Most women receive their contraceptive method from the private sector, although the public sector supplies the majority of LARCs and injectables ^19^. Use of the private sector appears to be more common in the South than in the North ^20^.

### Qualitative assessment

We conducted in-depth interviews (IDIs) with four providers in each of the five programs currently piloting LNG-IUS introduction in Nigeria ( Table 2), for a total of 20 providers. For each program, we obtained lists of sites/providers from program managers and purposively selected providers from two regions, except for UCHI which has a single site. Regions were selected to provide representation from the North and the South of Nigeria while keeping in mind practical constraints linked to the coverage of the different programs and to visiting sites for interviews.

**Table 2.** Key characteristics of the five introduction programs.

	MSION ^a^	Rotary	DKT	UCHI	SFH
Channel	Social franchising program	Public sector service delivery (secondary facilities)	Direct marketed sales to private clinics	Public sector service delivery (tertiary hospital)	Social franchising program
LNG-IUS product currently utilized ^b^	Donated ICA product	Donated ICA product	Eloira (commercial product)	Donated ICA product	Donated ICA product
Price structure	1,500-3,000 Naira (USD 4-8)	Free	Set by provider	Free	3,000 Naira (USD 8)
Geographic Coverage ^b^	17 states	8 states	Varies	One facility	18 states

^a^ Although MSION introduced the LNG-IUS through mobile outreach teams, social franchise clinics, and public sector providers, only social franchise clinics are covered in this assessment. ^b^ As of July 2019. MSION, Marie Stopes International Organisation Nigeria; ROTARY, Rotary International; DKT, DKT International; UCHI, University College Hospital Ibadan; SFH, Society for Family Health; LNG-IUS, levonorgestrel intrauterine system; ICA, International Contraceptive Access Foundation.

Program managers directly informed providers or provided letters of introduction to facilitate contacts. Potential participants were approached by phone to schedule interviews. Three local, Masters-level, female research assistants hired as consultants interviewed providers in English individually and in private at health facilities in July 2018. Each provider was interviewed once, using topic guides covering training, clinical experience with the LNG-IUS, perceived advantages and disadvantages of the method, and resources and activities for supporting service delivery (see *Extended data*) ^22^. At the end of the interview, the research assistants used structured questions to elicit time estimates for completing certain tasks. IDIs were audio-recorded, then transcribed and uploaded to NVivo 12 for coding and applied thematic analysis. Transcripts were verified against audio-recordings by a supervisor for accuracy and completeness and were not returned to participants for validation. Three analysts shared transcripts for coding, running periodic checks for intercoder agreement on 25% of transcripts. Codes included a priori codes identified based on informational needs and data-driven codes that emerged from the initial reading of transcripts. Analytic memos were prepared to explore patterns in the data, after which we used Excel matrices to summarize and compare the prevalence of key themes across the five programs.

### Costing assessment

The service delivery costing assessment was based on the introduction of the LNG-IUS with 71 trained providers in 40 social franchise clinics across 18 states in the SFH program between May 2017 and July 2019. The SFH program was selected for the costing exercise partly because of convenience (SFH is a partner on the broader research project that funded this assessment) and also because SFH is looking to introduce a new LNG-IUS product, Avibela, in 2020 and insights could help inform a broader introduction strategy. 

***Calculation of incremental costs of introducing LNG-IUS in SFH pilot settings***

To estimate the costs of service delivery in SFH-supported clinics during the pilot phase, we used an Excel-based costing template (see *Extended data*) ^22^ to collect input on the resources required for method provision, and on the associated unit and total costs. We included costs of direct labor from health care perspective for counseling, insertions and removals; costs of consumable supplies (except where costs were negligible including for antiseptic, soap, and iodine); costs of insertion/removal kits; and costs for provider training. Labor costs for method provision were informed by time estimates for counseling, insertion and removal collected through a questionnaire administered to all SFH providers (n=39) participating in a refresher training on the LNG-IUS in April 2018 (see Table 3).

**Table 3.** Provider estimates of time spent on different tasks from questionnaire administered to Society for Family Health (SFH) providers and average monthly salary for nurse-midwives (N=39).

	Time, min	
**Counseling a new FP client**		
Mean	23	
Range	7-60	
	Time inserting, min ^a^	Time removing, min ^a^
**Method provision and removal**		
LNG-IUS		
Mean	9	4
Range	4-30	1-5
Copper IUD		
Mean	10	4
Range	3-40	1-8
One-rod implant		
Mean	7	14
Range	2-25	2-30
Two-rod implant		
Mean	11	21
Range	3-38	3-50
**Average monthly salary for** **nurse-midwife in SFH network**	US $273	

Note: The midpoint was used when providers gave a range. ^a^ For insertions and removals, providers were asked to estimate time from when a woman lays down on the table to when she stands up.

The IUD/IUS kits were comprised of 13 re-usable instruments required for insertion and removal of either method; based on input from clinical experts, we assumed that each kit could be used for 500 insertions/removals ( Table 4 and Table 5). Given that the LNG-IUS is currently donated to the SFH program, we did not include any associated cost for the LNG-IUS commodities for this component of the analysis.

**Table 4.** Estimated price of equipment per insertion/removal for intrauterine devices (IUDs) and implants.

	Price (USD)	Approximate number of procedures (lifespan of supplies)	Price per insertion or removal
IUD Insertion and Removal Kit ^a^	$51.52	500	$0.10
Implant Insertion and Removal ^b^	$14.92	500	$0.03

Sources: a – Personal communication with Society for Family Health/Nigeria, 2019. b - As cited in: https://www.ghspjournal.org/content/ghsp/4/Supplement_2/S83.full.pdf.

**Table 5.** Estimated price of consumables for IUDs, implants and injectables.

Method	Sterile gloves	Sharps box	Lidocaine	5 ml Syringe	Scalpel Blade	Sterile Drape	Sanitary Pads	Sterile Gauze Sponge	Adhesive tape
Implants	$0.29	$0.01	$0.64	$0.04	$0.07	$0.25	n/a	n/a	$0.01
IUD/LNG-IUS	$0.29	n/a	n/a	n/a	n/a	$0.25	$0.11	$0.25	n/a
Injectable	$0.29	$0.01	n/a	$0.04	n/a	n/a	n/a	n/a	n/a

Sources for table: As cited in: https://www.ghspjournal.org/content/ghsp/4/Supplement_2/S83.full.pdf IUD, intrauterine device; LNG-IUS, levonorgestrel intrauterine system.

SFH staff calculated costs for provider trainings (training of master trainers, initial cascade trainings with providers, and refresher training with providers) based on program expenditure reports. For demand creation, SFH used a provider-initiated awareness generation model. This involved the provider carrying out talks about contraception with women in the facilities who came for post-natal or child wellness visits. In these contexts, the provider would include the LNG-IUS in the context of a full method mix. As such, there was no incremental cost for demand creation as part of routine service delivery. Likewise, routine clinical supervision with LNG-IUS providers was conducted as part of supportive supervision that would have occurred anyway, so there was no incremental cost associated for ongoing clinical supervision for the LNG-IUS. Additional information about training and demand-generation activities are described in Table 6.

**Table 6.** Components of SFH LNG-IUS pilot included in the costing exercise.

	Description
**Provider training** **model**	First a centralized “training of trainers” session was convened by SFH. The two-day session was led by three expert clinicians with eight master trainers who participated. Next, nine “cascade” training sessions were held around the country with a total of 71 providers trained who delivered services at 40 clinics. Cascade trainings took place through on-site trainings at clinics. Each training session lasted two days; the first day involved a didactic lecture, and the second day included hands-on practice sessions. During the didactic lectures, providers learned about method characteristics and how to insert the LNG-IUS using a model. On the second day, providers practice insertion with actual clients. Approximately one year later, refresher training sessions were held at six central locations, with providers traveling off-site to participate; these refresher trainings took place at both clinics and off-site locations. The two-day refresher trainings included providers who had been previously trained as well as providers who were new to offering the LNG-IUS (with a focus on training new providers in clinics where previously trained providers were no longer employed/ available).
**Demand creation**	In all clinics supported by SFH, a provider-initiated demand creation model was used. This involved the provider carrying out demand generation and awareness talks with women in facilities who came for postnatal or child wellness visits. Some of these providers also conduct regular community mobilization events. Information about the LNG-IUS was included in the group talks as part of a broader method mix. Therefore, there was no incremental cost for demand creation as reflected in Table 9.
**Supportive** **supervision**	Supervisory support visits are conducted by SFH on regular basis in order to ensure high quality service provision and to conduct on-the-job coaching for the providers in order to enhance proficiency and confidence in counseling and service provision. This is completed for all methods including the LNG-IUS. Therefore, there was no incremental cost for supportive supervision as reflected in Table 9.

SFH, Society for Family Health; LNG-IUS, levonorgestrel intrauterine system.

SFH staff provided service statistics on the numbers of LNG-IUS inserted and removed during the assessment timeframe (May 2017 through July 2019). We then calculated the total incremental direct cost of LNG-IUS introduction and divided by the total number of LNG-IUS insertions (1,949) to obtain a cost per LNG-IUS insertion.

***Calculation of direct cost per CYP for all family planning methods***

For the broader cost per CYP analysis comparing the direct service delivery costs of the LNG-IUS to those of other family planning methods in Nigeria, we used commodity costs to international procurers for all methods (taken from the UNFPA catalogue or, for the LNG-IUS, provided by Medicines360, the supplier of Avibela, which is the new, more affordable commercial product that is being registered by SFH in Nigeria). Although many family planning programs in Nigeria including SFH receive most of their contraceptive commodities for free by donation (typically from UNFPA or USAID), we wanted to include the commodity prices in this part of the analysis to better represent the economic cost to the health system.

To calculate direct service delivery costs per CYP, we followed a similar approach to that described for an earlier analysis in Kenya ^23^. Briefly, the analysis included commodity costs as described in Table 7, as well as costs of consumable supplies, estimated costs of instruments per client visit, and costs of direct labor for counseling, insertion, removal, and resupply if required for each method. We then used standard CYP conversion factors for each method ^24^. For short- and mid-acting methods, we aggregated costs of visits made throughout the year to achieve one CYP. For long-acting methods, we divided the costs by the appropriate conversion factor. Following current guidance, we used a conversion factor of 3.3 years for the LNG-IUS (assuming the method is labeled for five years of use). Consistent with previous analyses, the direct cost per CYP calculation does not include provider training costs because it assumes a steady state once providers have been oriented and trained on all family planning methods.

**Table 7.** Commodity prices included in cost per CYP assessment *.

Oral contraceptives	$ 0.25
Copper IUD	$ 0.32
Depo Provera	$ 0.93
Sayana Press	$ 0.85
Female condom	$ 0.40
Implanon	$ 8.50
Jadelle	$ 8.50
Levoplant	$ 6.90
Avibela LNG-IUS	$15.00
Male condom	$ 0.03

* Costs obtained from UNFPA Procurement Catalogue except for Avibela. Avibela prices provided through personal communication with supplier, Medicines360, 2018. CYP, couple years protection; IUD, intrauterine device; LNG-IUS, levonorgestrel intrauterine system; UNFPA, United Nations Population Fund.

### Ethical statement

The National Health Research Ethics Committee of Nigeria approved this assessment (NHREC/01/01/2007). This activity was also reviewed by FHI 360’s Protection of Human Subject Committee (PHSC) in the United States and deemed to be exempt from ethical approval because it was not human subjects research (1192089). Written informed consent to participate and to audio-record the interview was obtained from all providers.

## Results

### Qualitative assessment

Provider IDIs lasted 27 minutes on average. Four MSION providers were replaced from the original pool of selected providers because facilities from the initial list were no longer in the MSION franchise network. Four replacements were also selected for DKT because providers had not yet offered the LNG-IUS product, and one because the trained provider had left the facility. Table 8 shows provider characteristics. Most providers were women, although all DKT providers were men. All providers had several years of experience providing the copper IUD. Average experience offering the LNG-IUS ranged from six months for UCHI providers to over five years for DKT providers (which likely refers to earlier experience with Mirena). Providers had varying levels of experience inserting the LNG-IUS. About half of all providers had removed an LNG-IUS at some point.

**Table 8.** Characteristics of providers in the qualitative assessment sample.

	MSN (N=4)	ROTARY (N=4)	DKT (N=4)	UCHI ^a^ (N=4)	SFH (N=4)	ALL (N=20)
Sex, n Male Female	2 2	0 4	4 0	1 3	1 3	8 12
Age, years	54	45	53	51	45	49
Experience offering contraceptives, years	19	15	18	20	7	16
Experience offering IUCD, years	19	15	18	19	6	15
Experience offering LNG-IUS, months	14	28	66	6	22	27
LNG-IUS insertions performed 1-5 6-20 Over 20	2 2 0	0 0 4	2 2 0	0 1 3	1 2 1	5 7 8
Ever done LNG-IUS removal ^a^ Yes No	3 1	3 1	2 2	3 0	0 4	11 8

^a^ Information not available for one UCHI provider. MSN, Marie Stopes International Organisation Nigeria; ROTARY, Rotary International; DKT, DKT International; UCHI, University College Hospital Ibadan; SFH, Society for Family Health; IUCD, intrauterine contraceptive device; LNG-IUS, levonorgestrel intrauterine system.

Findings from interviews with providers are presented in three main areas: perspectives on the LNG-IUS; capacity and resources; and other aspects of product introduction.

### Providers’ perspectives on the LNG-IUS

Providers were aware of the differences between the LNG-IUS and the copper IUD, including different durations of action, different mechanisms of action, and different bleeding profiles. Most providers highlighted the fact that the LNG-IUS did not cause heavy bleeding as an advantage of the method over the copper IUD, with several providers also mentioning reduced cramping. Two providers explicitly said that the LNG-IUS was a better method than the copper IUD.

Approximately two-thirds of providers appreciated the clinical benefits of the LNG-IUS, especially for women with heavy bleeding or fibroids but also for endometriosis and anemia. Several providers mentioned that their LNG-IUS clients did not experience any side effects, and two providers explicitly noted minimal side effects as an advantage over other hormonal methods.

*The health benefit that I said, reduction in bleeding, very, very good in correcting bleeding and even anemia, it even reduces the length of your menstrual cycle, if you are bleeding for 5 days, it reduces to 3 which is better, if you’re bleeding more, there’s tendency for anemia. So, the health benefits really, really outpower the other ones like the copper T [531]*

Most providers commented favorably on reduced bleeding and amenorrhea as consequences of method use. While several providers reported that their clients generally accepted bleeding changes with proper counseling, some providers noted mixed acceptability of amenorrhea among users. Several providers mentioned lifestyle benefits associated with reduced bleeding, including buying and using less sanitary pads and minimal interference with sexual activity.

Providers themselves did not appear to have concerns about the LNG-IUS as a method, and about half said that they typically did not receive any complaints from LNG-IUS users. However, several providers noted complaints by clients about initial spotting and at least two providers said they conducted pregnancy tests to reassure amenorrhoeic clients. A few providers also reported contraindication for women with active uterine infections as a disadvantage.

*One [disadvantage] is the issue of prolonged spotting, which is common to most of our clients at the initial period. They experience prolonged spotting, but since we already have that at the back of your mind, when we were counseling them, we prepare their minds, so psychologically they know what to expect and they are not panicky, and most times with time, it resolves without any treatment or management…some are so comfortable because of the amenorrhea, some will say “madam, I want to be seeing my blood every month, come and remove this thing.” They were counseled, and they knew, [but] they believe there is a dirty thing piling up in the system and there is no amount of counseling you can do, they will just say no, give me my copper, I want to be seeing my blood. [456]*

Some providers mentioned concerns around privacy and safety of uterine placement among women, and fears inherited from earlier myths about the copper IUD. A couple of providers in one program reported their clients’ general aversion to hormonal contraception.

With the exception of one provider who was not asked this question, all providers said they wanted to continue offering the LNG-IUS, although one had some reservations due to the amount of effort required at counseling to overcome fears and misperceptions. Reasons for wanting to continue offering the LNG-IUS included the non-contraceptive health benefits of the method, women liking the LNG-IUS, and expanding the method mix.

### Capacity and resources

While all providers were experienced offering the copper IUD, they had varying exposure to the LNG-IUS prior to its introduction in each program. For example, one female provider said she had been an LNG-IUS user herself for the past 25 years, a few providers had been introduced to Mirena in earlier trainings, and a couple of providers reported prior experience inserting LNG-IUS products with their clients without receiving formal training. In contrast, a few providers had only heard about the method in books and one provider said it was new to them when they were trained.

Many providers indicated that the steps for inserting and removing the LNG-IUS were generally similar to those for the copper IUD, although they acknowledged small differences in the insertion process. One quarter of providers mentioned insertion challenges, including loading the first time and inserting in women with fibroids. Others reported no challenges, and one third of providers said they found loading easier with the LNG-IUS than with the copper IUD. Some providers found device placement more “technical” with the LNG-IUS because of the need to wait for the arms to open before pushing to the fundus.

When asked to estimate insertion times, 16 providers gave the same amount of time for the LNG-IUS as the copper IUD (around 11 minutes), three said that inserting the LNG-IUS was faster, and one that it took longer (results not shown but similar to those provided in Table 3). Most providers estimated that it took them more or the same amount of time to insert the LNG-IUS than a two-rod implant, and typically longer than to insert a one-rod implant. All providers who had already removed an LNG-IUS gave the same time estimate for removing the LNG-IUS and the copper IUD (six minutes). Removing the LNG-IUS took the same amount of time or less than removing a one-rod implant but was faster than removing a two-rod implant for most providers.

Over half of providers reported having sufficient equipment for LNG-IUS provision, while several other providers said they could manage but would benefit from additional equipment, and a couple of providers in one program reported that their instruments needed replacing due to repeat usage and decontamination. At least one provider in each of four programs indicated needing more consumables, and a few went on to explain that the costs of procuring consumables themselves were passed on to clients.

*We don’t have enough gloves and other [things] but we used to buy. That is why sometimes, we always ask the patient to buy small gloves when we don’t have, so we can make sure we control the prevention and infection control here. [232]*

All providers described having protocols in place for infection prevention, with the main examples provided being disinfecting and sterilizing equipment, but also handwashing and wearing gloves, generally maintaining an aseptic environment, and properly disposing of waste. All providers in one program emphasized screening women for active pelvic infections. Although providers were generally confident about their protocols, some reported challenges including time requirements for autoclaving and procurement of consumables.

*We have several protocols for handling of the instruments, but you can run them through HLD [high-level disinfection], dry them, autoclave. Some instruments can be run through soapy water, washed, dried, autoclaved…we do autoclaving almost 90% or 95% of the time. Sometimes [we use] most of the plastic instruments when we’re very busy, those we do HLD on them but the metal instruments, even after we do HLD, we still have to rinse and still autoclave. [141]*

### Other aspects of product introduction: Gaps in demand-generation, availability and pricing

Although providers typically talked about including all methods in their counseling to support informed choice by women, several providers said they recommended the LNG-IUS to women with heavy bleeding or fibroids, and a few others that they generally promoted the LNG-IUS more actively than the copper IUD, or in one case, implants, to their clients. One female provider who was an LNG-IUS user volunteered that she was using herself as an example with her clients to show the method did not cause any problems, while another provider said they showed empty LNG-IUS packs to prospective clients to convince them that other women were using the method.

Overall, providers observed that uptake of the LNG-IUS was limited to date, with a few providers saying that uptake of IUDs in general was low and several others noting that the LNG-IUS was currently less popular than the copper IUD. Most providers highlighted a general lack of awareness of the LNG-IUS. All providers in one program described an organized community outreach model that is used to generate demand and present the range of methods. Providers in other programs did not regularly engage in any regular outreach or education, with some reporting resource and staff constraints.

Recommended strategies to address the lack of awareness of the LNG-IUS included using the radio, posters and flyers or conducting health education in communities. Many providers also advised improving access by making the LNG-IUS available at more service delivery points, with a few providers noting that other places besides their organization or network that offered the method typically used Mirena, which was priced too high for women in their community.

*[To improve public awareness] we [need to] put them on the radio…they’ve not seen it and they’ve not seen people taking it. So they [need to] increase the awareness because very few of them when they come for family planning, you give them everything but before you even finish your counselling even they will already tell you what they want…[continuing later in the interview] The awareness can only be greater a little bit in the clinic but when people see it outside, they believe it more. It is a very good method but people don’t seem to like it maybe because they are not adequately informed about it [146]*

Pricing strategies varied across programs (see Table 2). Some providers reported charging a minimal fee for consumables or client cards in addition to the recommended price, and a couple of providers revealed they sometimes charged wealthier clients more in order to lower the price for poorer women. Many providers in programs charging a product fee felt that cost remained a barrier, despite being lower than the price for Mirena (the price of Mirena was cited by some providers at around 40,000-45,000 naira or 110-125 USD).

*The main problem is that the ones that are available to them are the commercially available [products]…As long as the cost is that high, it will be difficult to get more and more clients to use it out there…the only thing that can make people opt for it is if it can come at the same price as the ordinary copper T 380A. As long as the price is high so shall it be difficult to convince people to bring out money for it. [455]*

### Costing assessment

The estimated cost of LNG-IUS provision from May 2017 through July 2019 including training of 71 providers across 40 facilities and direct costs for labor, commodities and equipment was USD 66,300 with the LNG-IUS commodity donated for free by the ICA Foundation. During that period, 1,949 LNG-IUS units were inserted in the 40 clinics involved in the pilot; as such, the average per unit direct cost was USD 34 (see Table 9). Of the total service delivery costs, 92% (USD 61,530) was comprised of the training inclusive of the original training of trainers, cascade trainings to the 40 clinics and refresher trainings among previously trained and newly trained staff.

**Table 9.** Direct service delivery costs of introducing levonorgestrel intrauterine system (LNG-IUS) in 40 clinics supported by the Society for Family Health in USD (May 2017-July 2019).

Number of clinics where method offered	40
Total number of LNG-IUS offered May 2017 through July 2019	1,949
Total number of LNG-IUS removed May 2017 through July 2019	29
Average number of insertions per site (all sites)	49
**Costs (USD)**	
Training of trainers	6,711
Cascade trainings	23,549
Refresher trainings	31,269
Incremental demand creation costs	-
Direct labor for time for counseling, insertions, and removals	2,896
Consumables	1,949
Equipment (amortized)	201
Incremental supportive supervision costs	-
**Total costs all sites (USD)**	**66,357**
**Cost per LNG-IUS insertion (if LNG-IUS commodities donated) (USD)**	**34**

**Figure 1.**
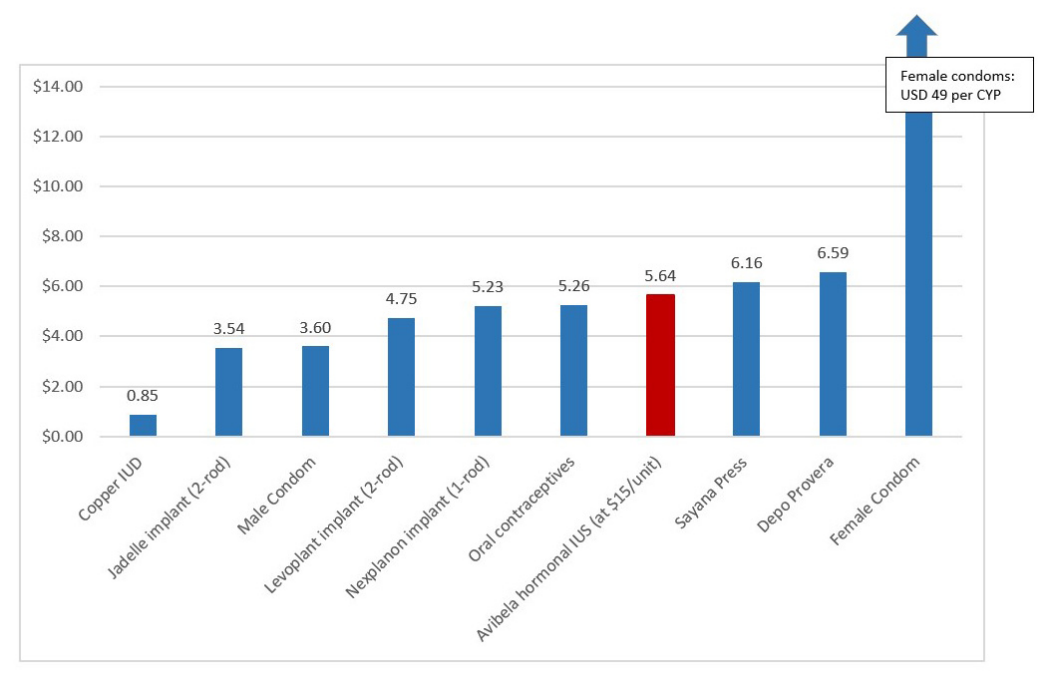
Direct service delivery costs per couple years of protection (CYP) in USD for various family planning methods.

The direct delivery costs per CYP are presented in Figure 1. The cost per CYP of the Avibela LNG-IUS (USD 5.64) is slightly higher than that of the one-rod implant, Nexplanon (USD 5.23), and is slightly lower cost per CYP than injectable methods (USD 6.16 for Sayana Press and USD 6.59 for Depo Provera). Injectables are currently the most commonly used method in Nigeria.

## Discussion

Our findings indicate that introduction of the LNG-IUS has been relatively well-received at the provider level. Providers saw the LNG-IUS as a clearly distinct product from the copper IUD and a valuable addition to the method mix. They were particularly appreciative of the therapeutic benefits of the LNG-IUS and of its reduced bleeding profile. Table 10 provides a summary providers’ perspectives on key barriers and facilitators affecting introduction of the LNG-IUS that were identified in this assessment.

**Table 10.** Summary of barriers and facilitators to further introduction of the LNG-IUS based on providers’ perspectives and experiences. Items in **bold** were cited in half of more of IDI respondents.

	Facilitators	Barriers
**Provider attitudes towards** **LNG-IUS**	**No heavy bleeding compared to copper IUD** **Therapeutic benefits for fibroids, anemia and** **endometriosis** **Treatment of menorrhagia** **Reduced bleeding or amenorrhea** Few systemic side effects Reduced cramping compared to copper IUD	Initial spotting Dislike or fear of uterine placement among some clients Resistance to amenorrhea among some clients Aversion toward hormonal contraception among some clients Not suitable when active infections
**Factors affecting quality** **of care**	**No major challenges with insertion and** **removal Confidence in privacy arrangements** **in current clinical setting** **Availability of equipment** **Confidence in infection prevention measures**	Procurement of consumables Equipment wear and tear Time for equipment processing Stepped up counseling
**System-level** **considerations**	Lower price than Mirena Affordability of method (in programs charging little or nothing)	**Limited awareness of method** High price compared to other LARCs Limited availability of method Staff and resource constraints to demand creation

LNG-IUS, levonorgestrel intrauterine system; IDI, in-depth interview; IUD, intrauterine device; LARC, long-acting reversible contraception.

These results are consistent with those from earlier research in Nigeria, which found that providers did not see the copper IUD and LNG-IUS as interchangeable products on several counts and identified reduced bleeding as a key non-contraceptive benefit ^14^. However, while providers in our assessment acknowledged the value of minimizing uterine bleeding, the most compelling method attribute does not seem to be the lifestyle benefits of lighter or shorter periods (or possibly amenorrhea) to their clients but, rather, the potential to avoid or control heavy, prolonged bleeding. This finding dovetails with a recent systematic review of contraceptive-induced bleeding changes reporting that heavy or prolonged bleeding is often poorly tolerated by women ^25^.

Providers’ positive perspectives towards the LNG-IUS were reinforced by the fact that they reported receiving few complaints from clients due to acceptability of bleeding changes with proper counseling and few side effects. However, our findings also point at some areas that may benefit from additional counseling or messaging to women and communities in order to increase method uptake, including resistance to amenorrhea and dislikes and fears related to hormonal contraception and uterine placement.

Providers all had prior experience with the copper IUD but varying experience with the LNG-IUS prior to its introduction in their respective programs. Overall, providers appeared to be comfortable with both the insertion and removal process, believed they had the proper equipment, and reported that they followed protocols for infection prevention and control. One potential area of improvement could be procurement of consumables, which impacts the ability to implement safety measures and to keep the price low, as providers otherwise tend to pass the cost on to their clients.

One consideration when introducing a new method is the effect it may have on the existing pattern of services. Although time estimates indicate that IUDs (inclusive of the LNG-IUS and copper IUD) are quicker to remove than implants, our findings suggest there may be some up-front opportunity costs in terms of insertion times, which could be further compounded by the need for more in-depth counseling in the context of a method that is not yet well-known and some aspects of which are prone to misperceptions. At the same time, shorter removal times for the LNG-IUS could make it attractive from a client, provider and/or health systems perspective, given that increased attention is being paid to the requirements for ensuring high-quality removal services and associated costs ^26, 27^.

Some important findings in this assessment have to do with broader constraints to LNG-IUS uptake, including limited availability and awareness of the method and a comparatively high price relative to other LARCs in programs where the method was not made available for free. To date, outside of the programs highlighted in this paper, LNG-IUS products are only available on a limited basis at a high cost in Nigeria. Providers’ input suggests increasing access to the LNG-IUS is likely to require a multi-pronged approach including further reducing price barriers, while programs simultaneously focus not only on provider training and high-quality service provision but also on demand creation inclusive of non-contraceptive benefits and differences between the LNG-IUS and the copper IUD. This conclusion is also supported by a recent assessment of the LNG-IUS conducted in Nigeria ^14^.

This is the first assessment we are aware of that includes a calculation of the incremental service delivery costs of introducing the LNG-IUS in a pilot setting in a developing country context. Up-front training costs were included in the calculation. However, because the per-insertion cost calculation included here is only based on the number of insertions over the first two-year time period of LNG-IUS introduction, this amount will likely not reflect the per-insertion cost as the LNG-IUS program becomes more mature (e.g. as more insertions are conducted over time) and as economies of scale are achieved. Nevertheless, this data from a pilot setting may be valuable for program managers and other decision-makers who are contemplating similar pilot introductions in other regions or settings in Nigeria. 

The estimated cost per insertion of USD 34 is in-line with other similar costing assessments of pilot LARC programs conducted in the region. For example, a recent analysis in Rwanda of a new program for postpartum IUD and postpartum implant insertion calculated an estimate per insertion cost of USD 25 for postpartum IUDs and USD 77 per implant, although in that case, the analysis included the cost of the commodities ^28^. Currently, SFH receives donated LNG-IUS commodities in Nigeria, but if the program becomes responsible for buying the LNG-IUS in the future, the cost to the program will increase. A new LNG-IUS product, Avibela, received notification of national regulatory approval in December 2019. At approximately USD 15 per unit (assuming an order of 100,000 units), the procurement price for Avibela will be substantially lower than that of other commercial LNG-IUS products currently on the market ( Table 1); however, at the USD 15/unit price, Avibela will still be approximately twice as much as the commodity price for contraceptive implants (which can be purchased for USD 6.90 - 8.50 per unit, depending on the implant) ^7, 29^. Given these considerations, as well as the feedback from providers about the importance of affordable pricing to clients, it will be important for the government in Nigeria and international donors to continue to seek strategies to further increase the affordability of the method. Also, the fact that 92% of the estimated cost of service delivery during the pilot phase was dedicated to provider training suggests that it will be important for service delivery groups to identify more affordable training models if and when the method is taken to scale. Finally, there were no dedicated resources to support targeted demand generation for the LNG-IUS in the SFH-supported clinics. Moving forward, investments in awareness generation will be required to increase awareness of and demand for the method among women, as reflected in the input received from the provider interviews.

As decision-makers consider if and when to make further investments in scaling up the LNG-IUS, it is important to note that even at a price point of USD 15 for the LNG-IUS commodity, our analysis shows that the direct service delivery costs per CYP of the LNG-IUS is similar to other commonly used family planning methods available in Nigeria including injectable contraception. This finding is aligned with a comparable analysis conducted in Kenya ^23^, and the results demonstrate that because long-acting methods including the LNG-IUS do not require regular resupply, they can be more affordable over time to health programs even if there is a higher up-front commodity cost that for short-acting methods. Furthermore, the CYP factor that is currently assigned to the LNG-IUS (3.3 CYP) is lower than the CYP factor that is currently assigned to the two-rod hormonal implant, Jadelle (3.8 CYP) ^24^. This is despite the fact that both methods are effective for up to five years. Given that there is emerging body of evidence regarding hormonal IUS use in LMICs including continuation rates ^30^, the authors recommend that the assigned CYP factor for the LNG-IUS be re-evaluated. If the CYP factor for the LNG-IUS were more similar to the CYP factor for Jadelle, this would further improve the cost per CYP of the LNG-IUS compared to other methods.

## Limitations

Despite the qualitative design and small sample size for the provider interviews, some generalizability of our findings is supported by commonalities in the thematic structure of results across multiple programs. However, there are also limitations to our assessment. All five projects that have supported LNG-IUS pilots introduced the method by providers who were already experienced with the copper IUD in programs providing high volumes of LARCs and typically operating in urban and peri-urban settings. The applicability of these findings may be limited in more typical service delivery conditions, as use of the copper IUD is below 1% in many sub-Saharan African countries ^31^. Time estimates for inserting and removing LARCs are self-reported and may not be accurate but provide useful directional information for the purposes of comparisons between the LNG-IUS and other methods. The cost assessment only focused on early costs of a pilot introduction in clinics supported by the SFH program; additional cost assessments are needed to evaluate the cost of routine service delivery in other settings and once providers have been trained and awareness of the method in the community has increased. To address some of these limitations, additional analyses are underway to examine the cost and cost-effectiveness of introducing the LNG-IUS in Nigeria.

## Conclusion

Providers’ positive experiences with the LNG-IUS and the fact that direct service delivery costs per CYP align with those for other methods suggest that the LNG-IUS could be an important addition to the method mix in Nigeria. The qualitative assessment of providers’ perspectives offers a reminder that effective product introduction strategies need to be multi-pronged and address both the supply and the demand side, as well as consider appropriate pricing of the LNG-IUS relative to other LARCs. The results presented in this paper may be useful to decision-makers including government officials, program managers and donors who are trying to make decisions about if and when to invest in additional introduction of the LNG-IUS within Nigeria and in other similar contexts.

## Data availability

### Underlying data

Full qualitative transcripts are not available for ethical reasons because even after removing directly identifiable information such as names and addresses, participant identity may be difficult to fully conceal, and research locations may remain potentially identifiable, presenting a risk of deductive disclosure. However, topic guides, codebooks and relevant excerpts of transcripts are available from the authors on reasonable request. Requests should be sent to the corresponding author at krademacher@fhi360.org or to familyplanning@fhi360.org. Requests will be granted to researchers for the purposes of comparative analysis, upon approval from relevant ethics committees.

### Extended data

Harvard Dataverse: Provision of the levonorgestrel intrauterine system in Nigeria. https://doi.org/10.7910/DVN/7JMETP ^22^

This project contains the following extended data:

- NigeriaLNGIUSAssessment_IDIguide_Providers_v.1.0.docx (in-depth interview guide)
- NigeriaLNGIUSAssessment_ICF_Provider-IDI_v1.0.docx (participant information and informed consent form)
- Nigeria_LNGIUS.Provision_Costing_template.xlsx (costing template)

Data are available under the terms of the Creative Commons Zero "No rights reserved" data waiver (CC0 1.0 Public domain dedication).
