# Supplementary material for: Provision of the levonorgestrel intrauterine system in Nigeria: Provider perspectives and service delivery costs
Source: Gates Open Res. 2020 Aug 6;4:119. [Version 1] doi: 10.12688/gatesopenres.13135.1 (PMC7463110; doi:10.12688/gatesopenres.13135.1)
Supplement: Peer review history

# Peer review: Reviewer response for version 1

**DOI:** 10.21956/gatesopenres.14322.r29338

**Published:** 2020-09-01

**Authors:** Jennifer Kidwell Drake

**Affiliations:** 1 PATH, Seattle, WA, USA

This is an excellent article summarizing provider perspectives and cost data from limited LNG-IUS introduction in Nigeria, and is positioned to make a valuable contribution to the literature. I suggest some revisions along the following lines: 

 A few details: 

1. First reference to Table 1, there's a missing word (I think Nigeria?). In Table 1, it would be optimal to know the price for all LNG-IUS options if available (and if not available, acknowledge that?). Final point - would it be possible to add a brief overview of product availability across FP 2020 countries? That might make the article more applicable for a wider range of readers.
2. Please justify/explain why you chose to focus specifically on the service delivery/health systems costs and not the broader societal costs (i.e., costs to women), which are also important for decision-makers to understand. Are there existing studies re: costs for women for IUD insertion that you could borrow from/reference (or even implants as a parallel)?
3. In the intro or conclusion is it worth at least summarizing/referencing client perspectives in FP 2020 countries re: the LNG-IUS or IUD/IUS in general? I recognize this is out of scope to address directly in your methodology/results, but helpful context to acknowledge.
4. Methods, under Qualitative Assessment - how did you determine sample size? 
5. Methods, under Costing Assessment - what is the source of the assumption that each kit could be used for 500 insertions or removals? (And assuming the slash stands for "or" and not "and", consider being explicit and just writing it out). 
6. For the trainings, helpful to note which training costs are/are not captured by program expenditure reports. Is provider time/salaries covered, for example?
7. Table 6 - can you share the frequency of visits under SFH supportive supervision model? 
8. Results - assuming the two providers who said that LNG-IUS was a better message said that spontaneously, without prompting. Maybe make that explicit? 
9. I think there may be a typo in one of the quotes - it says "...some are so comfortable because of the amenorrhea" but the quote makes it sound like it should be uncomfortable?
10. I am not familiar with the clinical term "loading" when you talk about insertion, might be helpful to define for other readers.
11. Also under Capacity and resources, you say, "one provider in each of four programs" - weren't there five programs in the study?
12. When you talk about pricing strategies, would be curious to hear if any providers shared more about clients' actual reaction to the price (not just general/hypothetical, as described)? 
13. For the Sayana Press cost per CYP, I assume this is for provider injections and not self-injection? Self-injection is being scaled in Nigeria, so important to note that distinction and the fact that you did not calculate cost per CYP for self-injection, which might (?) be lower given reduced visits (although could be offset by longer up-front counseling, potentially).This needs to be noted in the last paragraph of the discussion as well, when you talk about resupply. 
14. Table 10 is great, but not sure your opening Discussion statement acknowledges clearly enough the wide array of concerns expressed about health workers. Even a quick phrase acknowledging those would be nice.
15. Suggest acknowledging in the intro or conclusion that Nigeria is explicitly committed to introducing new methods and coordinating across introduction of new contraceptive methods, and if/how these type of data might help benefit the system and all products beyond just LNG introduction. Nigeria seems to be a leader along these lines and it would be nice to highlight.
16. In Limitations, you note that there were commonalities in thematic structure across multiple programs - was that clearly stated in Results before? Any notable exceptions to flag that might impact any points in your discussion?
17.  What about costing data availability, in terms of open access? That should be addressed explicitly.

Is the work clearly and accurately presented and does it cite the current literature?

Yes

If applicable, is the statistical analysis and its interpretation appropriate?

I cannot comment. A qualified statistician is required.

Are all the source data underlying the results available to ensure full reproducibility?

No

Is the study design appropriate and is the work technically sound?

Yes

Are the conclusions drawn adequately supported by the results?

Yes

Are sufficient details of methods and analysis provided to allow replication by others?

Yes

Reviewer Expertise:

My input falls under the category of "a number of small changes" rather than more significant ones. I have participated in several research studies focused on the introduction of DMPA-SC and self-injection, including costing studies, namely in Burkina Faso, Senegal, and Uganda.

I confirm that I have read this submission and believe that I have an appropriate level of expertise to confirm that it is of an acceptable scientific standard, however I have significant reservations, as outlined above.

# Peer review: Reviewer response for version 1

**DOI:** 10.21956/gatesopenres.14322.r29335

**Published:** 2020-09-01

**Authors:** Michael Mbizvo

**Affiliations:** 1 Population Council, Lusaka, Zambia

General:

 It is my opinion that the study succinctly presents findings from the qualitative assessment of LNG-IUS providers in Nigeria, which are complemented by a cost assessment. A general lack of awareness of LNG-IUS was observed. Findings reinforce the need to offer wider contraceptive method choices. It is noteworthy that costs were comparable with similar methods, while additional therapeutic benefits were reported. The paper thus adds to the evidence base on provider perspectives, especially in a context of low contraceptive uptake reported for Nigeria in general, and provider influences that may either  limit or facilitate contraceptive methods uptake in particular.

 Specific:

1. While the abstract results start with highlighting providers’ observations of how users accepted the “collateral” therapeutic benefit of the method, it is also important right at the outset to get insights on method acceptance for contraceptive benefit and insertion and removal processes. Thereafter, the added benefits could be alluded to.
2. In the third paragraph of the Introduction, where reference is made to “…with concerns among providers related to infections or risks of infertility…” I would propose adding “potential”, before infections. 
3. Under Methods, subsection “Setting”, first paragraph currently reads more like part on the study rationale. I would suggest that actual study contextual setting and health systems under which the study was conducted, be described.
4. I have given a “no” to availability of sources of data, with reference to qualitative information, which is in line with the explanation given by the authors to delink data provided with identity of respondents.      

Is the work clearly and accurately presented and does it cite the current literature?

Yes

If applicable, is the statistical analysis and its interpretation appropriate?

Not applicable

Are all the source data underlying the results available to ensure full reproducibility?

No

Is the study design appropriate and is the work technically sound?

Yes

Are the conclusions drawn adequately supported by the results?

Yes

Are sufficient details of methods and analysis provided to allow replication by others?

Yes

Reviewer Expertise:

NA

I confirm that I have read this submission and believe that I have an appropriate level of expertise to confirm that it is of an acceptable scientific standard.

# Peer review: Reviewer response for version 1

**DOI:** 10.21956/gatesopenres.14322.r29336

**Published:** 2020-08-25

**Authors:** Gathari Ndirangu Gichuhi

**Affiliations:** 1 Pathfinder International, Nairobi, Kenya

The authors demonstrate the high acceptability by health providers in Nigeria to counsel for and offer the hormonal IUS to FP clients in sites that have been offering this method as part of a menu of short-acting and long-acting reversible contraceptive methods. Further, the article shows that while the cost per CYP of the hormonal IUS in Nigeria is similar to that of the contraceptive implants but much higher than that of the Cu-T IUD, this cost is generally similar to that of other commonly used contraceptive methods. It is instructive to note that the cost is lower than that of the three-monthly injectable contraceptive (DMPA and DMPA-SC). Considering the fact that this method has significant non-contraceptive benefits to users and is indicated as a therapeutic agent for women with heavy menstrual bleeding from a variety of non-cancerous causes that include uterine leiomyoma, adenomyosis, and endometrial hyperplasia expanding its access to women in low and middle-income countries through price reduction will transfer huge benefits to the women. 

 The article is logically presented, clearly describing the methods used and data management, and lays out the results well, with sensible discussion and conclusion. Although the authors do not avail the source data due to concerns of the inability to completely de-identify the transcripts, they offer relevant excerpts upon request to the corresponding author. 

 I'd suggest the following few edits;

 Consistent abbreviation of Marie Stopes International Organization Nigeria. In Table 8, it is abbreviated as MSN while in other sections, the authors refer to it as MSION.

 Under methods (setting), it would be helpful to point out what the disparities look like, other than merely mentioning that there's " *more limited use of modern contraception in*" Northern Nigeria. This description probably sits better in the introduction. 

 In Table 2, it is unclear what it means to have the pricing structure *set by* the *provider* in the DKT sites. Additional details would be helpful, even as a note to describe the table. One would expect that providers would charge for this service within some set limits. 

 In Table 5, sterile gauze sponge is indicated as not applicable (n/a) for provision of the contraceptive implant yet one would expect that the arm is cleaned with an antiseptic using sterile gauze as part of infection prevention and control. Clarity on this will be helpful. 

 Under results, this verbatim quote of a health provider on infection prevention and control is unclear and the authors could improve what they're communicating by editing it;

*We don’t have enough gloves and other [things] but we used to buy. That is why **sometimes, we always** ask the patient to buy small gloves when we don’t have, **so we can make sure we control the prevention and infection control here**. [232].*

 In Table 10, the cell that lists facilitators of quality of care needs editing;

**No major challenges with insertion and**

***removal Confidence in privacy arrangements***

***in current clinical setting***

**Availability of equipment**

**Confidence in infection prevention measures**

 In the same table, the last factor on barriers for provider attitudes towards LNG-IUS is incomplete;

*Not suitable when active infections*

 The references are relevant and recent. 

 I appreciate the opportunity to have reviewed this article that I believe will contribute to the body of knowledge regarding expanding access to the hormonal IUS to women who cannot afford the current commercial rates.

Is the work clearly and accurately presented and does it cite the current literature?

Yes

If applicable, is the statistical analysis and its interpretation appropriate?

Yes

Are all the source data underlying the results available to ensure full reproducibility?

Partly

Is the study design appropriate and is the work technically sound?

Yes

Are the conclusions drawn adequately supported by the results?

Yes

Are sufficient details of methods and analysis provided to allow replication by others?

Yes

Reviewer Expertise:

Family planning and contraception; maternal and newborn health; sexual and reproductive health; public health; obstetrics and gynecology.

I confirm that I have read this submission and believe that I have an appropriate level of expertise to confirm that it is of an acceptable scientific standard.
